# Effect of weightbearing and foot positioning on 3D distal tibiofibular joint parameters

**DOI:** 10.1038/s41598-022-12963-2

**Published:** 2022-06-07

**Authors:** Firas Souleiman, Martin Heilemann, Robert Hennings, Pierre Hepp, Boyko Gueorguiev, Geoff Richards, Georg Osterhoff, Dominic Gehweiler

**Affiliations:** 1 AO Research Institute Davos, Davos, Switzerland; 2 Department of Orthopaedics, Trauma and Plastic Surgery, University Hospital Leipzig, Leipzig, Germany; 3 ZESBO—Center for Research On Musculoskeletal Systems, Leipzig University, Leipzig, Germany

**Keywords:** Musculoskeletal system, Translational research, Medical research, Preclinical research, Orthopaedics

## Abstract

The aim of this study was to investigate the effect of different loading scenarios and foot positions on the configuration of the distal tibiofibular joint (DTFJ). Fourteen paired human cadaveric lower legs were mounted in a loading frame. Computed tomography scans were obtained in unloaded state (75 N) and single-leg loaded stand (700 N) of each specimen in five foot positions: neutral, 15° external rotation, 15° internal rotation, 20° dorsiflexion, and 20° plantarflexion. An automated three-dimensional measurement protocol was used to assess clear space (diastasis), translational angle (rotation), and vertical offset (fibular shortening) in each foot position and loading condition. Foot positioning had a significant effect on DTFJ configuration. Largest effects were related to clear space increase by 0.46 mm (SD 0.21 mm) in loaded dorsal flexion and translation angle of 2.36° (SD 1.03°) in loaded external rotation, both versus loaded neutral position. Loading had no effect on clear space and vertical offset in any position. Translation angle was significantly influenced under loading by − 0.81° (SD 0.69°) in internal rotation only. Foot positioning noticeably influences the measurements when evaluating DTFJ configuration. Loading seems to have no relevant effect on native ankles in neutral position.

## Introduction

Syndesmotic injuries are associated with sprain or fractures of the ankle in up to 18% or 45% of the cases, respectively ^1–4^. For the general athletic population, rates of 10–20% have been reported ^5^. Foot positioning is relevant for the pathomechanics of syndesmotic ankle injuries and appears to have a high impact on the distal tibiofibular joint (DTFJ) configuration, e.g. in dorsiflexion and external rotation injuries ^6–8^. Adequate reduction and stabilization are important to achieve a satisfying outcome with timely return to sport activities. To ensure anatomical reconstruction, identification of malpositioning is important. Numerous two-dimensional measurements for DTFJ assessment have been established ^9–13^. However, two-dimensional measurements represent a simplification of the geometry that is not justified considering the complexity of this joint ^14,15^. Therefore, three-dimensional (3D) measurement methods are becoming more popular as part of the technological development ^15–17^. Since the correct reduction of the fibula in the fibular notch of the tibia is the main factor for good functional long-term outcome and stability, accurate analysis of DTFJ is crucial ^18–22^.

Weightbearing cone-beam computed tomography (WBCT) is an alternative imaging technique allowing scanning under loading conditions with low radiation exposure ^23^. Initial studies using WBCT demonstrated promising results in terms of effectiveness in detecting syndesmosis injuries ^24^. Nevertheless, the benefit in the detection of syndesmotic lesions is discussed ^25–30^. Especially in the immediate post-traumatic and post-operative situation, the patients are unable to stand on the injured leg under full weightbearing because of pain. The systematic influence of foot position on DTFJ configuration in case of intact ligaments has not been sufficiently investigated ^31,32^. It is well known that bilateral imaging should be used for good DTFJ assessment due to interindividual differences ^9,15,33^. However, what is beneficial when using bilateral imaging if the position of both feet is different? Are there any systematic errors due to particular non-neutral foot positions? Therefore, the aim of this study was to investigate the effect and magnitude of different loading scenarios and foot positions on DTFJ configuration. It was hypothesized that both foot positioning and loading have a significant effect on this configuration despite intact ligaments.

## Materials and methods

**Figure 1:**
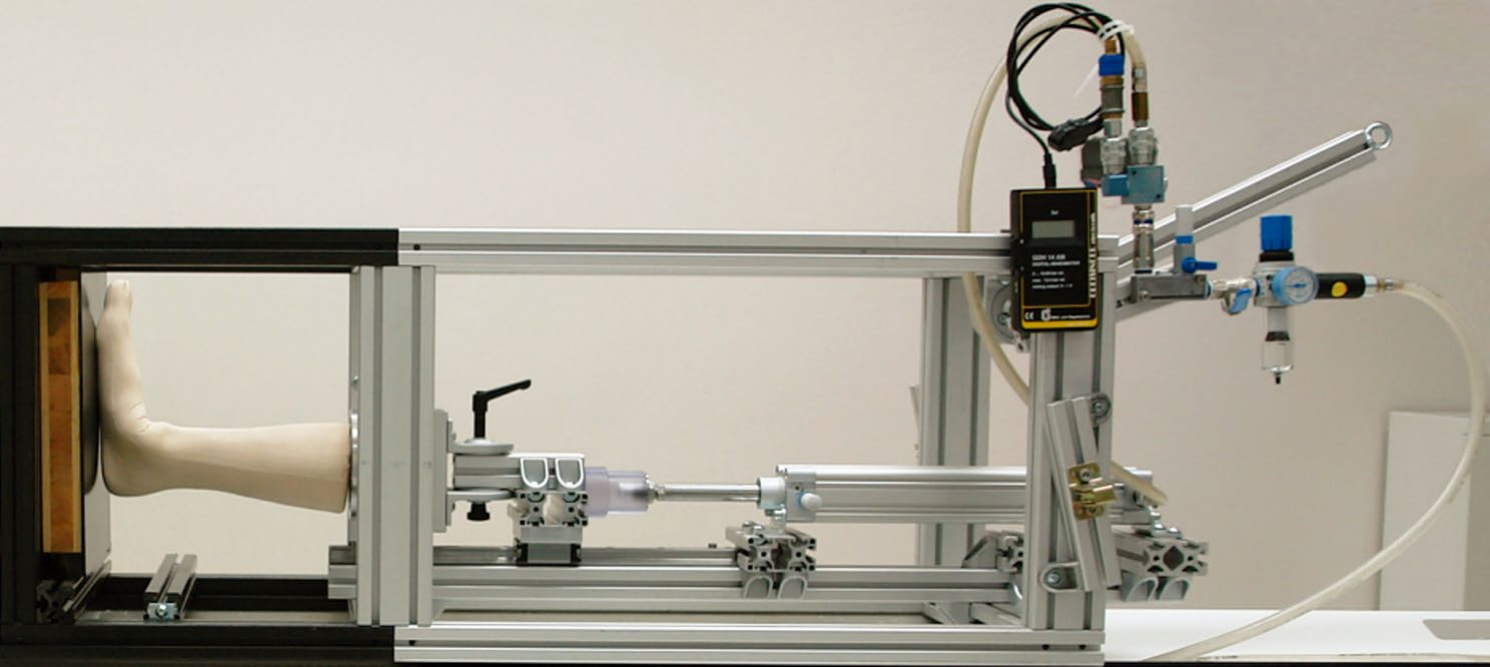
Custom-made loading frame with an artificial lower leg mounted for CT scanning under weightbearing in neutral foot position. The distal end of the frame is made out of radiolucent composite material, the main part is made out of aluminum. A pneumatic cylinder is connected to a compressed air system at the proximal end of the frame.

Fourteen paired fresh-frozen (− 20°C) human cadaveric lower legs from four male and three female donors aged 81 years on average (range 59–91 years) with no visible preexisting pathology, trauma or surgery were used. After completion of the entire study, the integrity of ligamentous structures (syndesmotic ligaments) was surgically confirmed under visual control. The specimens were defrosted 24 h before preparation, then cut and embedded at the level of the middle tibia below the tuberosity in polymethylmethacrylate (PMMA; SCS-Beracryl, Suter-Kunststoffe AG, Fraubrunnen, Switzerland) with intact syndesmotic ligaments and membrana interossei. The fibula was cut at the level of embedding to be excluded from fixation. Each specimen was mounted horizontally in an air pressure-controlled radiolucent frame, specifically designed for positioning and axial loading of human cadaveric lower legs during computed tomography (CT) scanning (Fig. 1) ^34^.

**Figure 2:**
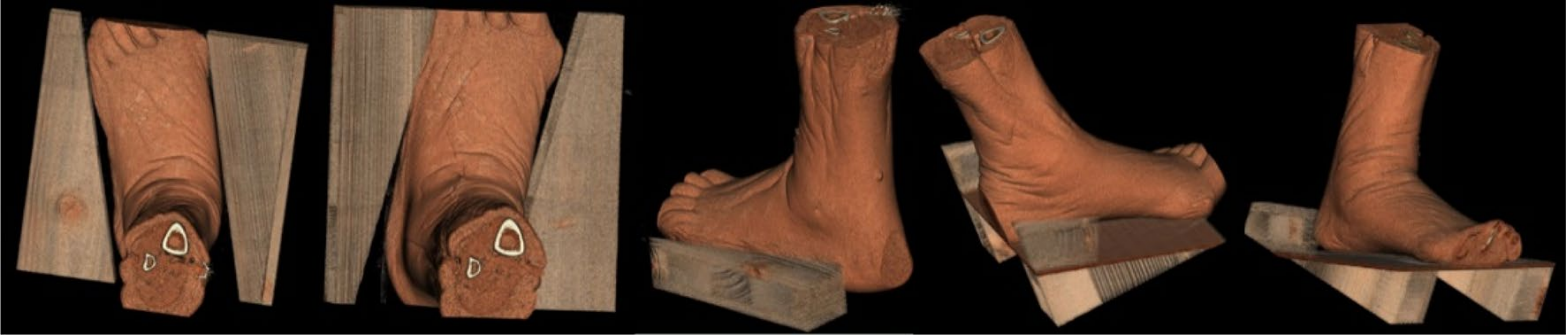
Wooden shanks used to maneuver the specimens in five foot positions in the loading frame during CT scanning; left to right: 15° external rotation, 15° internal rotation, neutral position, 20° plantar flexion, 20° dorsal flexion. The exemplified images are processed via volume rendering using Mimics Innovation Suite (Materialise, Leuven, Belgium).

The specimens were fixed in five different foot positions using wooden shanks—neutral (NP), 15° external rotation (ER), 15° internal rotation (IR), 20° dorsiflexion (DF), and 20° plantar flexion (PF) for CT scanning of each position in unloaded (75 N) and loaded (700 N) condition (Fig. 2). The two loading conditions are intended to simulate (1) unloaded single-leg stance—where only muscle forces are applied—and (2) fully loaded single-leg stance of an average person ^34^. The CT scans were performed with a slice thickness of 0.63 mm (SOMATOM Emotion 6, Siemens Healthcare GmbH, Erlangen, Germany). Following CT scanning, the Digital Imaging and Communications in Medicine (DICOM) data was processed in 3D Standard Triangle Language (STL) parts of the tibiae and fibulae using a standardized segmentation protocol (Mimics Innovation Suite, Materialise, Leuven, Belgium).

The STL data sets of the tibiofibular joints were evaluated using Matlab software package (MathWorks, Natick, MA, USA). For best possible comparability, the z-axis of the coordinate system was realigned for each foot in neutral position by determination of the tibia axis. The tibia axis was determined by calculation of the centroids of two tibial cross sections, located 50 mm and 80 mm proximally to the tibial articular surface, and represented by their connecting line.

In order to evaluate the effect of foot positioning on ankle joint parameters under the same axial loading condition (75 N or 700 N), each tibiofibular dataset in non-neutral positions (ER, IR, DF and PF) was matched to the data of the corresponding foot in neutral position using rigid iterative closest point algorithm based on the respective tibial geometry. Consequently, alignment differences of the fibulae between the non-neutral and neutral foot positions became visible. These differences were quantitatively assessed by calculation of three 3D parameters defined in previous work: clear space difference (diastasis), vertical offset (fibular shortening), and translation angle (rotation) ^15^. The translation angle was based on the combined anteroposterior and mediolateral displacement of the fibula around the tibia.

**Figure 3:**
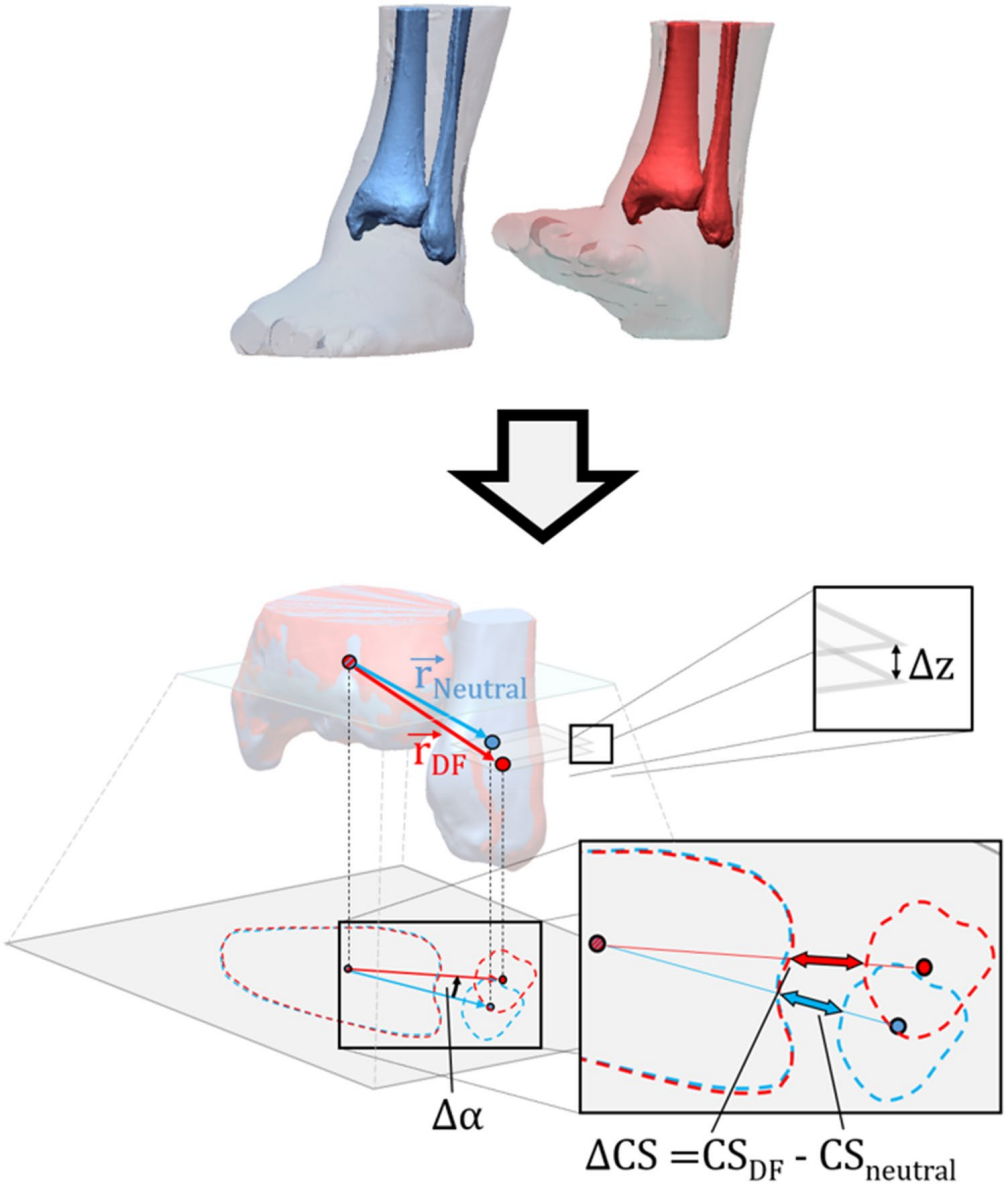
Matching (based on tibial geometry) and parameters calculation for an ankle in dorsal flexion relative to its neutral position. Clear space difference (∆CS), vertical offset (∆z) and translation angle (∆α) are determined by the connecting vectors (${\overrightarrow{\mathrm{r}}}_{\mathrm{Neutral}}$, ${\overrightarrow{\mathrm{r}}}_{\mathrm{DF}}$) of the respective tibial and fibular centroids. The illustration was made using Geomagic Design X software (3D Systems, Rock Hill, SC,USA) and Microsoft PowerPoint (Microsoft, Redmond, WA,USA).

The clear space difference ($\Delta CS$) between the corresponding tibiofibular joints was measured along the connecting line of the centroids of tibial and fibular cross sections located 10 mm proximal to the articular surface. The vertical offset ($\Delta z$) and translation angle ($\Delta \alpha$) were calculated from the connecting vector between tibial and fibular centers of volume. For calculation of the centers of volume, the tibia and fibula were virtually cut 20 mm proximal to the articular surface (distal tibial plafond), as the configuration of their distal ends is decisive for the anatomy of the tibiofibular joint. The translation angle mainly describes the anteroposterior translation of the fibula but can also be slightly influenced by mediolateral translation. Measurements of the vertical offset and translation angle on matched  ankles is schematically visualized in Fig. 3.

To evaluate the effect of weightbearing in each separate position, the data of each loaded specimen (700 N) was best fit matched to its unloaded data (75 N) in the same position, followed by the same 3D measurements.

All parameters of interest were tested for normal distribution with Lilliefors test. Subsequently, clear space difference, vertical offset and translation angle were tested for significance using Paired-Samples t-test with posthoc Holm-Bonferonni correction for multiple comparisons, since each parameter was tested for several foot positions and loading scenarios. Statistical analysis was performed at a level of significance 0.05 using Matlab.

### Ethics approval

All experiments were carried out under the relevant guidelines and regulations. Additionally, the internal review boards at Science Care (Phoenix, AZ, USA) and AO Research Institute Davos (Davos, Switzerland) approved the study. This investigation was performed in accordance with the ethical standards as laid down in the Declaration of Helsinki (1964) and its later amendments or comparable ethical standards.

### Consent to participate

All donors gave their informed consent inherent within the donation of the anatomical gift statement during their lifetime.

### Consent for publication

All donors gave their informed consent inherent within the donation of the anatomical gift statement during their lifetime.

## Results

**Figure 4:**
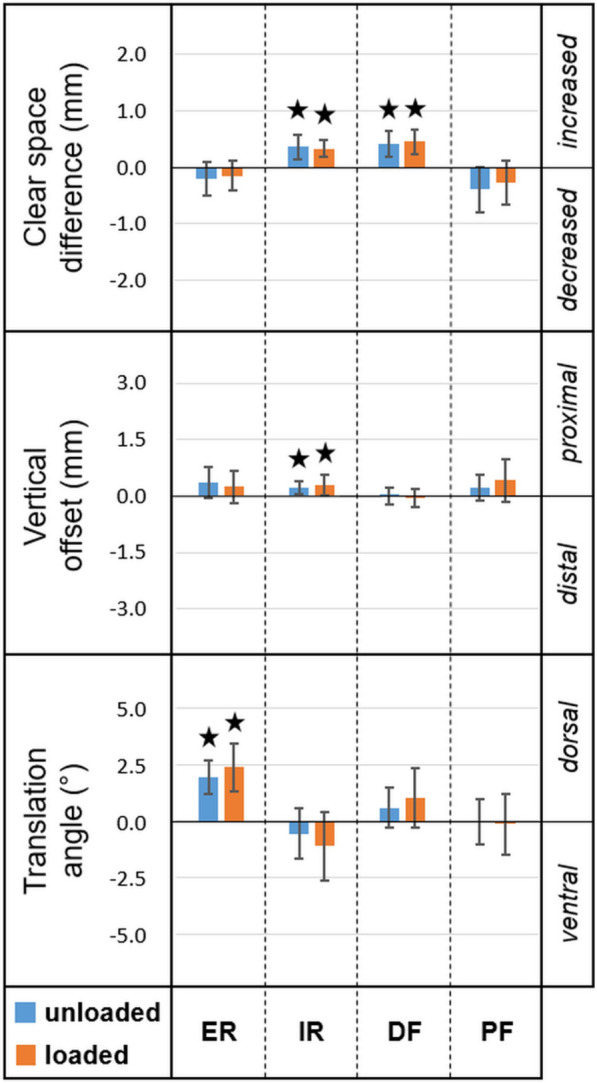
Clear space difference ($\Delta \mathrm{CS}$), vertical offset ($\Delta \mathrm{z}$), and translation angle ($\Delta \mathrm{\alpha }$) for each foot position relative to neutral position in either loaded or unloaded condition. Stars indicate significant differences. Axes are scaled to corresponding thresholds for native anatomy ($\Delta \mathrm{CS}: \pm 2\mathrm{ mm}, \Delta \mathrm{z}: \pm 3\mathrm{ mm}, \Delta \mathrm{\alpha }: \pm 5^\circ$) in order to visualize relative extent of the respective measures ^15^.

Figure 4 visualizes clear space difference, vertical offset and translation angle for internal and external rotation, as well as for dorsal and plantar flexion, all relative to the corresponding neutral position. Mean value, standard deviation (SD) and p-value for each separate evaluated foot position and loading scenario are presented in Table 1.

**Table 1:** Clear space (CS) difference, vertical offset, and translation angle in neutral position, external/internal rotation (ER/IR) and dorsal/plantar flexion (DF/PF), presented in terms of mean value and standard deviation (SD) for different loading scenarios together with the corresponding p-values from statistical comparisons.

Matched specimen		Reference specimen		CS difference (mm)			Vertical offset (mm)			Translation angle (°)		
Position	Loading scenario	Position	Loading scenario	mean	SD	P-value	mean	SD	P-value	mean	SD	P-value
ER	Unloaded	Neutral	Unloaded	− 0.20	0.30	0.540	0.35	0.41	0.177	1.96	0.75	< 0.001
ER	Loaded	Neutral	Loaded	− 0.15	0.26	0.968	0.24	0.43	0.968	2.36	1.03	< 0.001
IR	Unloaded	Neutral	Unloaded	0.36	0.22	0.001	0.21	0.18	0.019	− 0.58	1.12	> 0.999
IR	Loaded	Neutral	Loaded	0.33	0.15	< 0.001	0.29	0.26	0.038	− 1.12	1.50	0.374
DF	Unloaded	Neutral	Unloaded	0.42	0.23	< 0.001	0.01	0.23	> 0.999	0.58	0.90	0.586
DF	Loaded	Neutral	Loaded	0.46	0.21	< 0.001	− 0.06	0.25	> 0.999	1.01	1.32	0.341
PF	Unloaded	Neutral	Unloaded	− 0.40	0.40	0.077	0.22	0.33	0.563	− 0.03	0.43	> 0.999
PF	Loaded	Neutral	Loaded	− 0.26	0.39	0.562	0.41	0.57	0.449	− 0.15	1.34	> 0.999
Neutral	Loaded	Neutral	Unloaded	− 0.06	0.18	> 0.999	0.09	0.18	> 0.999	− 0.35	0.41	0.217
ER	Loaded	ER	Unloaded	0.03	0.12	> 0.999	0.01	0.18	> 0.999	0.12	0.49	> 0.999
IR	Loaded	IR	Unloaded	− 0.01	0.23	> 0.999	0.22	0.21	0.051	− 0.81	0.69	0.024
DF	Loaded	DF	Unloaded	− 0.08	0.19	> 0.999	0.09	0.18	> 0.999	0.16	0.62	> 0.999
PF	Loaded	PF	Unloaded	0.12	0.52	> 0.999	0.29	0.44	0.583	− 0.39	0.71	0.996

### Effect of foot position

The measurements demonstrated systematic errors due to particular foot positions compared to the neutral position for each of the three parameters of interest.

Clear space was significantly increased in IR (unloaded: p = 0.001, loaded: p < 0.001) and DF (unloaded: p < 0.001, loaded: p < 0.001). This parameter was most affected by DF under loading (mean difference: 0.46 mm, SD: 0.21 mm). ER and PF had no significant influences on the clear space (p ≥ 0.077).

Significant proximal vertical offset was caused by IR (unloaded: p = 0.019, loaded: p = 0.038). Although the largest vertical offset was observed for PF under loading (mean: 0.41 mm, SD: 0.57 mm), this did not represent a significant change (p = 0.449). No significant influence on vertical offset was detected for ER and DF (p ≥ 0.177).

Furthermore, ER caused significant translation of the fibula in dorsal direction (unloaded: p < 0.001, loaded: p < 0.001). Translation angle was most biased by ER under loading (mean: 2.36°, SD: 1.03°). No significant effect of foot position on translation angle was detected in IR, DF, and PF (p ≥ 0.341).

### Effect of loading

The comparisons of each separate foot position under loading versus the same position without loading resulted in no significant clear space difference (p > 0.999) and vertical offset (p ≥ 0.061) among all specimens. Only in IR loading resulted in a significant translation angle (mean: − 0.81°, SD: 0.69°, p = 0.024).

## Discussion

In this study, CT imaging of human cadaveric lower legs revealed a significant effect of the foot position on clear space difference, vertical offset and translation angle, resulting in systematic errors of these DTFJ measures caused by non-neutral foot positioning during CT imaging. For this reason, the hypothesis that foot positioning considerably influences the DTFJ configuration with intact syndesmotic ligaments is confirmed. In contrast, the hypothesis that loading has an overall significant effect should be rejected.

In order estimate the extent of the systematic errors related to foot positioning, the outcomes of the current study need to be compared with thresholds for native anatomy. Native thresholds for clear space difference (± 2 mm), vertical offset (± 3 mm) and translation angle (± 5°) were determined in previous work using the same 3D measurement method and are consistent with other findings in the literature ^9,15,35–37^. Comparing the parameter changes of the presented study to these thresholds, the translation angle was the most biased parameter influenced by foot positioning. A mean of 2.36° in dorsal direction for ER under loading corresponded to 47% of the threshold value. The difference for mean translation angle between ER and IR under loading was even 3.48°, accounting for 70% of the native threshold. Furthermore, the measured clear space increase of 0.46 mm on average for DF under loading corresponded to 23% of the respective threshold value. Comparing the mean values for unloaded DF and PF, clear space differed by 0.82 mm equaling to 41% of the threshold. These large relative changes related to the thresholds for native anatomy demonstrated that rotation and flexion highly influence the fibula position along the frontal and sagittal axis. The current findings are consistent with a previous clinical study using mortise view radiographs to examine the medial clear space with respect to the foot position, where the authors reported significantly different syndesmosis widths between neutral position and different phases of plantar flexion ^31^. An unloaded four-dimensional CT analysis could show similar effects of foot positioning ^33^. Changes in diastasis and fibular rotation were emphasized there, whereas the current work could additionally crystallize changes in sagittal translation.

The current study identified vertical offset explicitly in proximal direction. Only IR resulted in significant vertical offset change accounting for only 10% and 7% of the corresponding native threshold for this parameter in loaded and unloaded condition, respectively. Therefore, the vertical alignment of the fibula seems to be more stable against rotation and flexion than its alignment in the horizontal plane.

Furthermore, the 3D matching and evaluation of the lower legs in loaded and unloaded condition revealed almost no influence of loading on the clear space and vertical offset in the current study. Only in IR loading lead to a significant translation angle, however, the change accounted for only 16% of the native threshold, representing a much smaller effect than foot positioning. Such a minor influence of loading was also reported in a recent human cadaveric study designed with different severities of syndesmotic injury ^30^. In contrast, a meta analysis could demonstrate that imaging under loading can relevantly support detection of syndesmotic injury when the syndesmotic region is examined in detail ^38^. These contradictory findings confirm that further investigations focusing on well-selected parameters of interest are necessary. Three-dimensional positional control could provide crucial scientific benefits in this field too.

Overall, the current results revealed considerable influence of foot positioning on the tibiofibular parameters, whereas no relevant effect of loading on the clear space and vertical offset was observed, in particular for neutrally positioned ankles. Similarly, a human cadaveric study with injured syndesmotic ligaments also assessed the influence of axial loading and external rotation on the DTFJ configuration, reporting a considerable influence of external rotation compared to axial loading ^39^.

In recent years, the clinical use of WBCT for investigation of foot and ankle injuries has gained popularity ^40^. At a first glance, the advantages seem obvious with lower radiation exposure, faster feasibility (in case of available equipment), weightbearing and thus more physiological imaging ^40,41^. However, the benefit for assessment of the DTFJ configuration has to be questioned more critically due to the reported little influence of weightbearing. It remains questionable whether the small effect of weightbearing—even in injured state—results from the loading itself or is due to issues related to the used measurement methods ^30^. There is evidence that 3D position control is superior to previous two-dimensional measurements because the former better identifies the complexity of a joint malalignment ^14–17,42^. A good measurement response could already be proven for patients with disorders of the forefoot, midfoot, and hindfoot ^40^. Clinical studies using WBCT imaging in combination with 3D measurements to assess DTFJ configuration in injured state of the syndesmotic ligaments are currently pending. These might demonstrate a greater influence of weightbearing examinations than in the current study performed in intact specimen state ^40^. Corresponding 3D reference values for uninjured and injured state of the DTFJ exclusively in neutral foot position have to be determined. Such examinations were previously performed without consideration of the foot position ^15^.

The results of this study demonstrate that foot positioning has a significantly greater influence on measured 3D parameters than loading in case of intact syndesmotic ligaments. This influence, including its quantitative dependence on foot positioning and loading, calculated by automatic 3D position control, has not been comprehensively elaborated to such an extent in any previous study. Changes of up to 70% of the native reference values in case of foot rotation are very high and could falsely influence the postoperative control. It has to be kept in mind that the internal and external rotation in the current study was limited to 15°. It is possible that a higher rotation could have resulted in even larger effects. In order to eliminate this error, the authors advocate the uniform use of a splint in neutral position. This is a cost-effective optimization of the measurement assessment and will lead to more comparable measurement values in the future. Especially in high performance athletes, who—as reported—are often affected by syndesmotic injuries, no mistakes should happen due to an incorrect measurement assessment. Numerous studies have described radiological malpositioning after surgical treatment of syndesmosis injuries ^18,20,43–45^. In retrospect, the imaging conditions under which the CT scans were performed must be analyzed.

To the best authors' knowledge, this is the first experimental study investigating in such detail the influence of rotation and flexion of the foot, as well as of weightbearing on the DTFJ configuration in case of intact syndesmotic ligaments. The current work has some limitations similar to those inherent to all cadaveric studies. A limited number of specimens was used. Static loads in constrained foot positions do not represent the complexity of human gait. It is possible that there are dynamic benefits of weightbearing recordings that could not be captured in the static examinations. Since this is a human cadaveric study, effects related to the lack of muscle activities cannot be excluded. Furthermore, for image acquisition in the CT frame it was necessary to perform a horizontal force application to the specimens. It can be assumed that some minor differences to the physiological stance are present. Furthermore, during preparation the tibia was resected at mid-shaft level, thus cutting off the proximal tibiofibular joint and excluding the fibula from the embedding. As a result, no direct force was applied to the fibula, which does not fully correspond to physiological conditions.

A major advantage of this work is the combination of CT scans in a loading frame, the use of a standardized segmentation protocol to create 3D bone models of the tibia and fibula, and an automated measurement procedure to calculate 3D parameters on the tibiofibular joint without rater-dependent detection of several landmarks. As the authors recommend the use of this beneficial measurement method in the future, the knowledge of the specific influence of foot position and loading, provided by this study, is essential.

In conclusion, a noticeable influence of foot positioning on the 3D DTFJ configuration was demonstrated using a automatic protocol. The loading seems to have no relevant effect on native lower legs in neutral position. Future studies could use the same 3D parameters to systematically investigate the effects of foot positioning and loading in case of syndesmotic ligament or other lesions of the foot and ankle.

## Data Availability

The datasets used and/or analyzed during the current study are available from the corresponding author on reasonable request.

## Code availability

The code used during the current study are available from the corresponding author on reasonable request.
